# Supplementary figures and images for: Ectopic expression of Aspergillus flavus uricase and URAT1 in therapeutic cells promotes intracellular degradation of uric acid in hyperuricemic mice
Source: PLoS One. 2026 Apr 20;21(4):e0347534. doi: 10.1371/journal.pone.0347534 (PMC13094949; doi:10.1371/journal.pone.0347534)

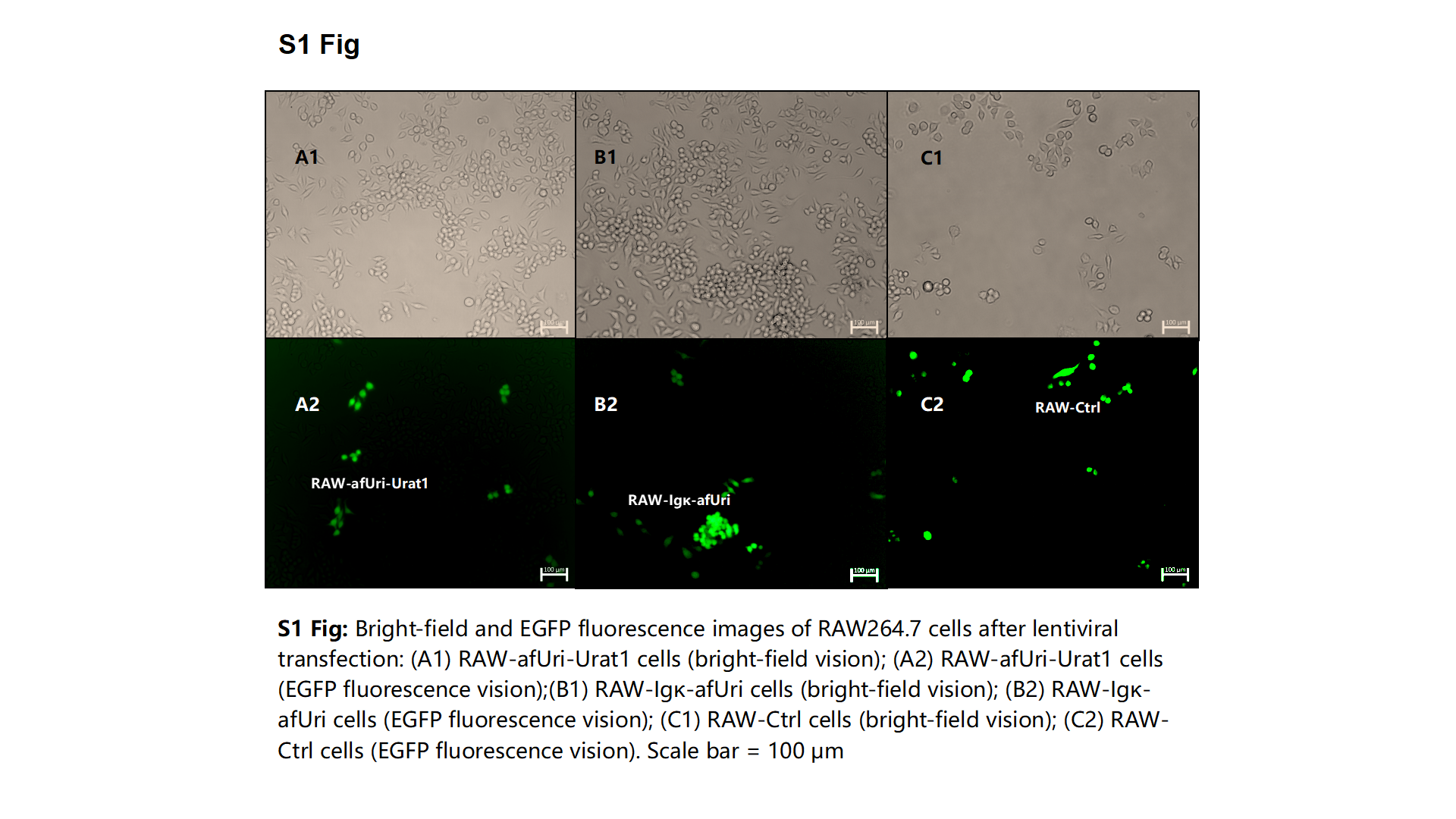

Supplement: S1 Fig — (TIF) [file pone.0347534.s001.tif]

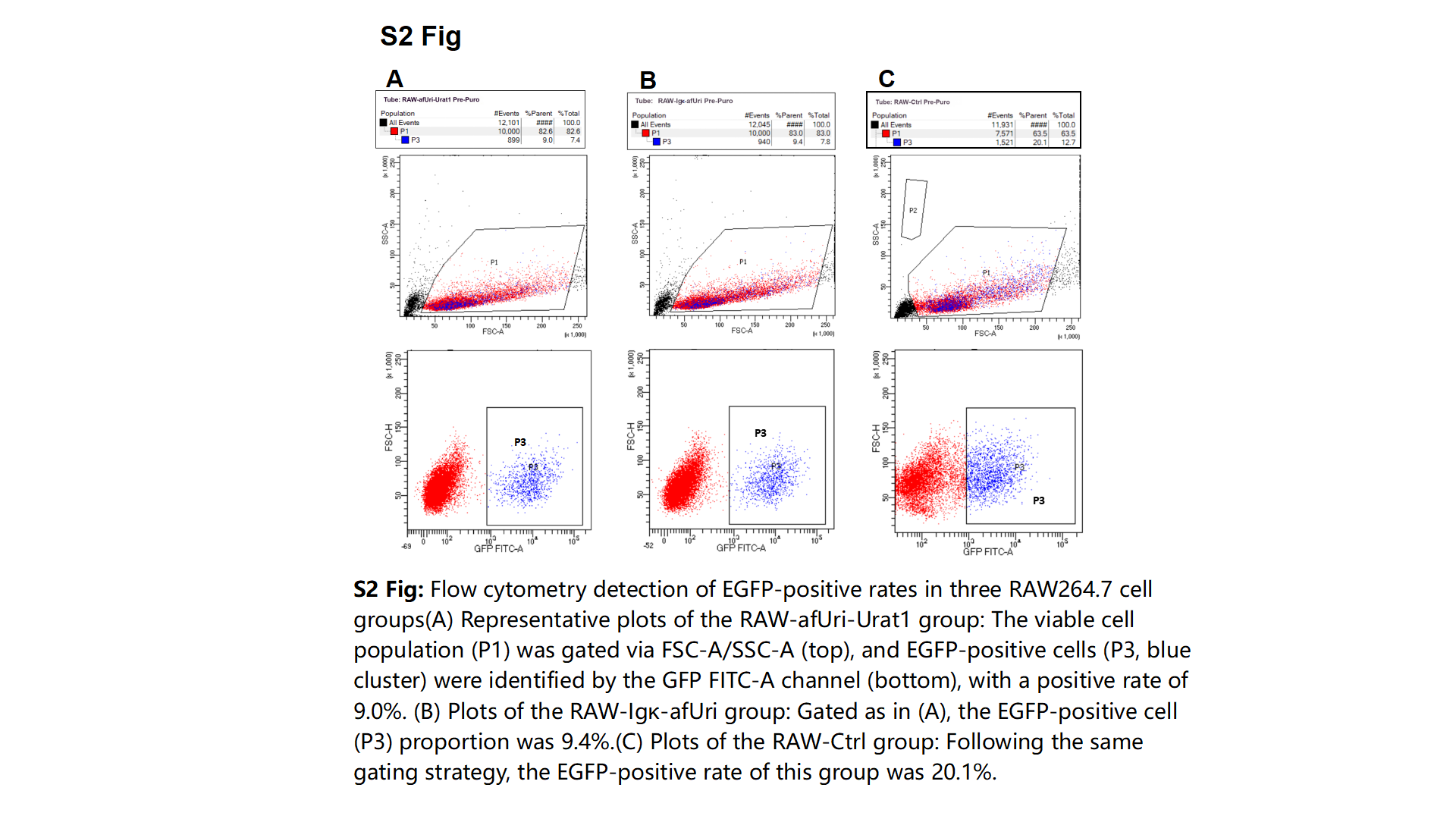

Supplement: S2 Fig — (TIF) [file pone.0347534.s002.tif]

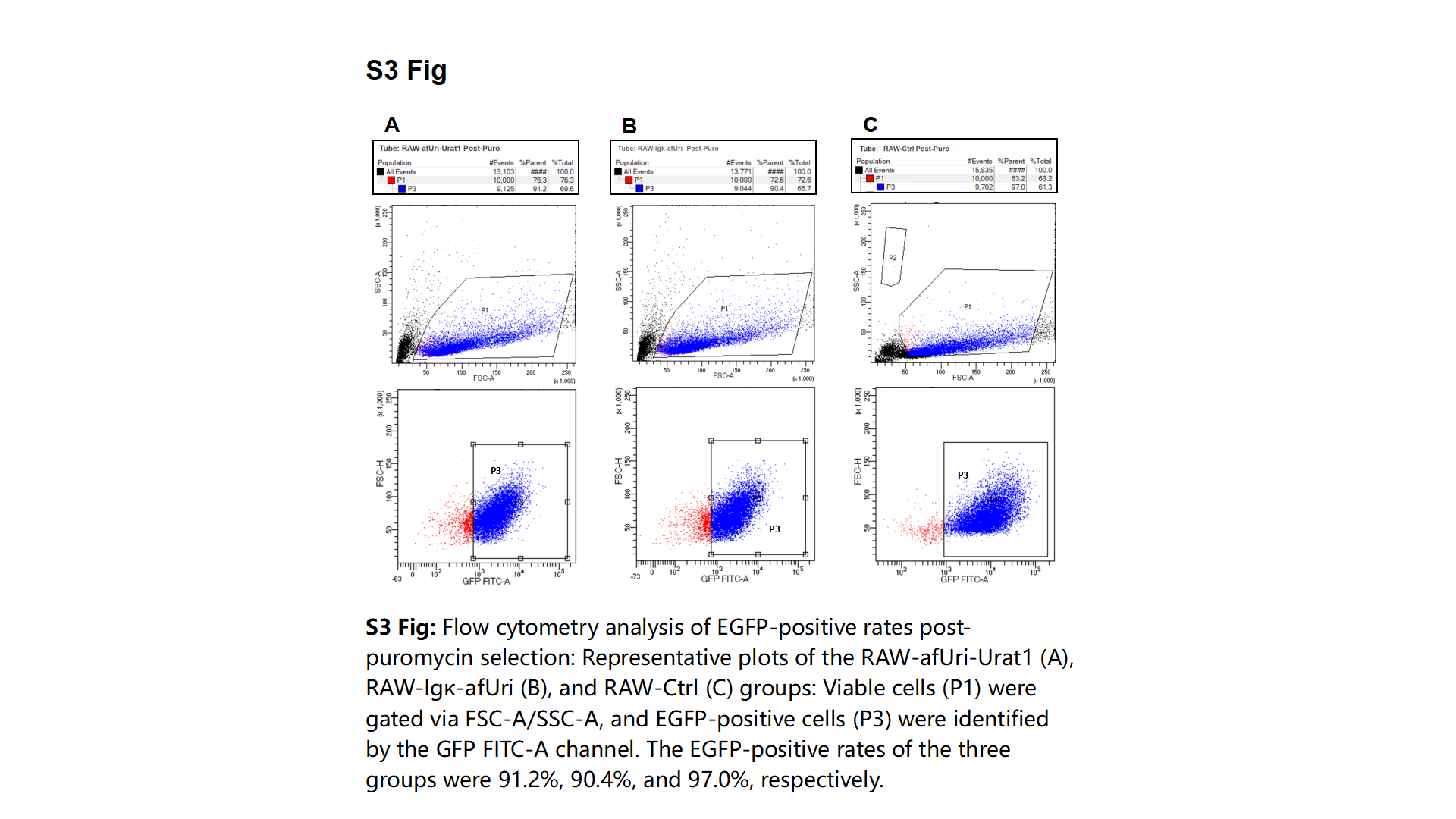

Supplement: S3 Fig — (TIF) [file pone.0347534.s003.tif]

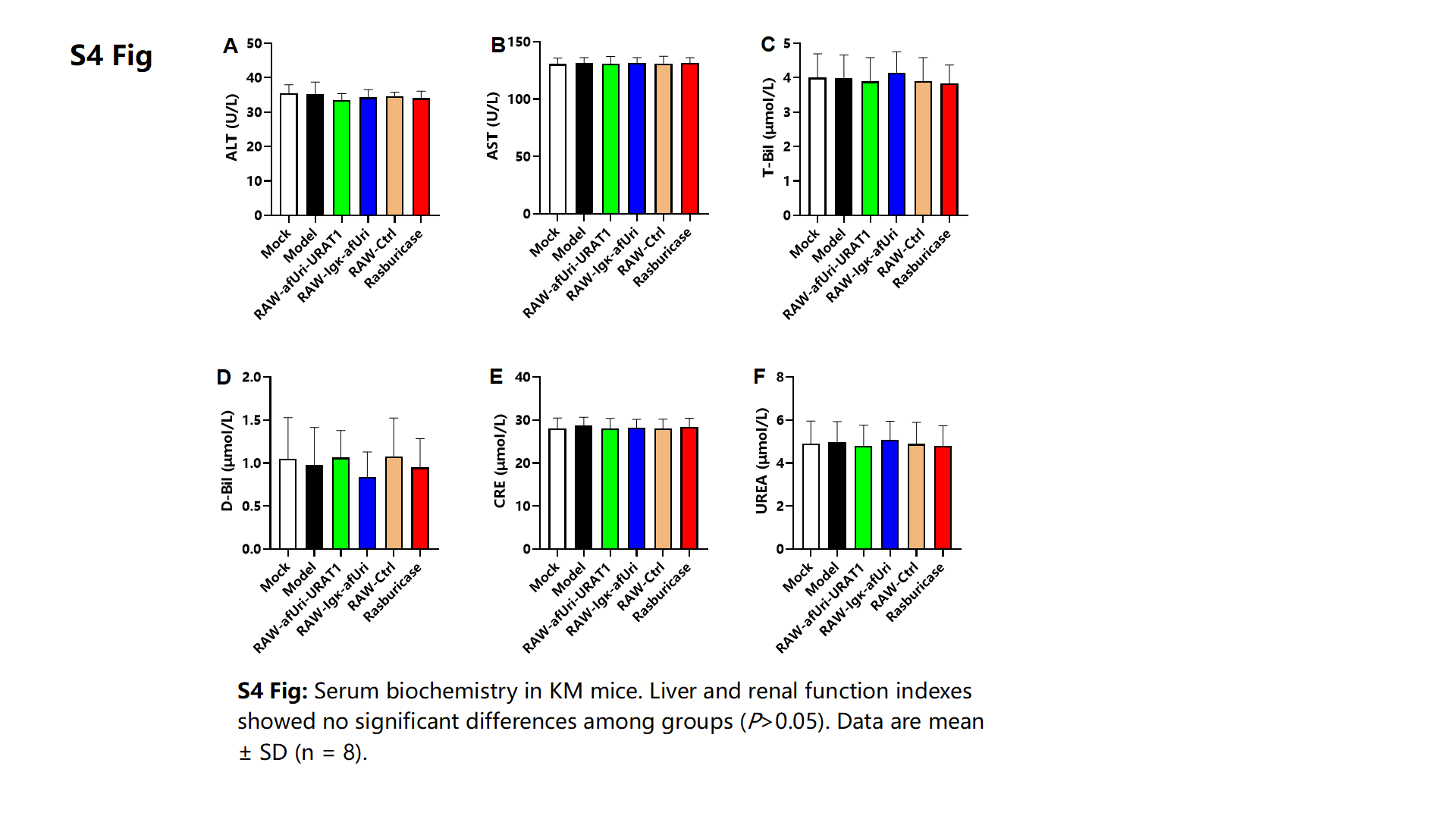

Supplement: S4 Fig — (TIF) [file pone.0347534.s004.tif]

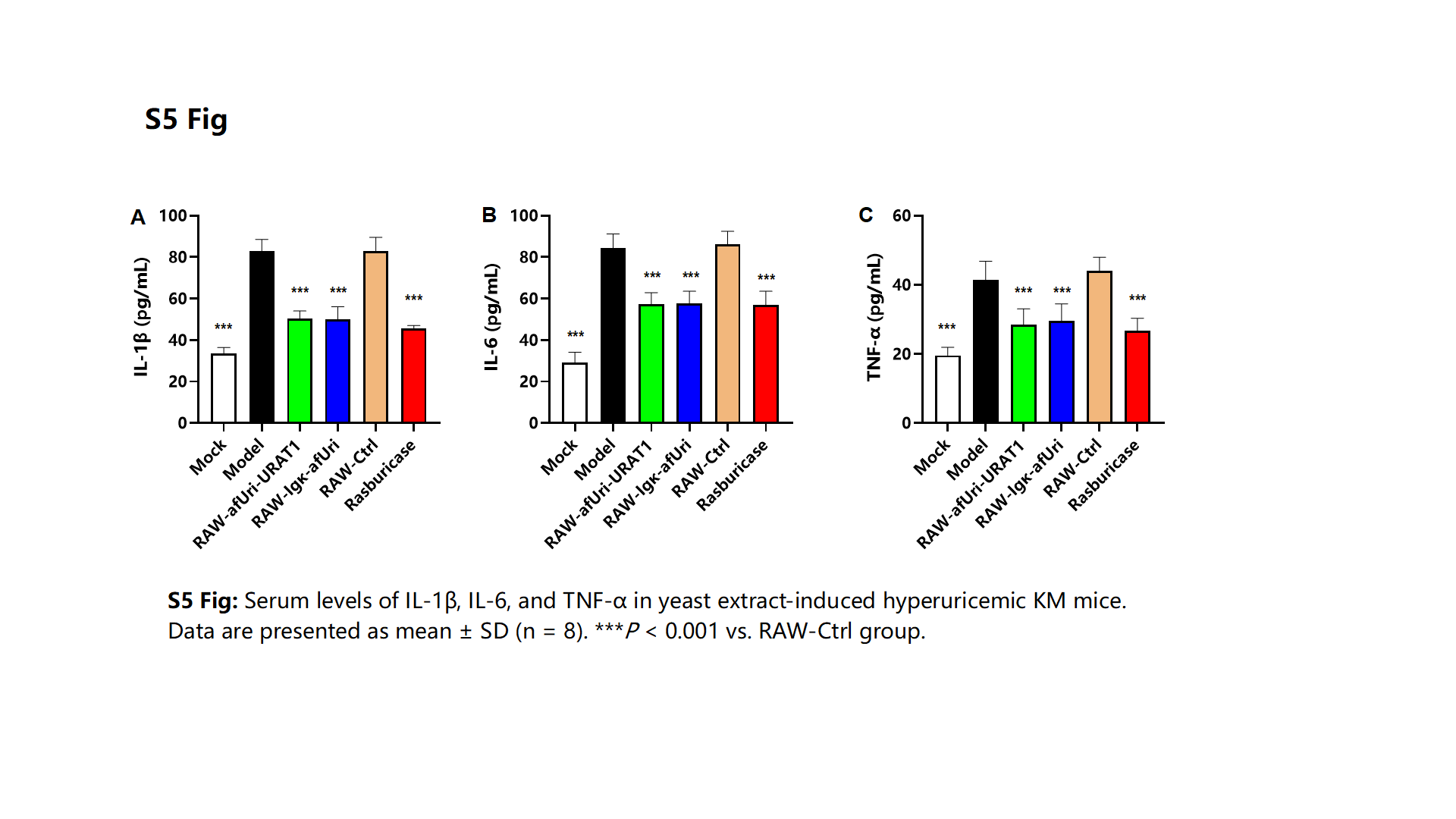

Supplement: S5 Fig — (TIF) [file pone.0347534.s005.tif]

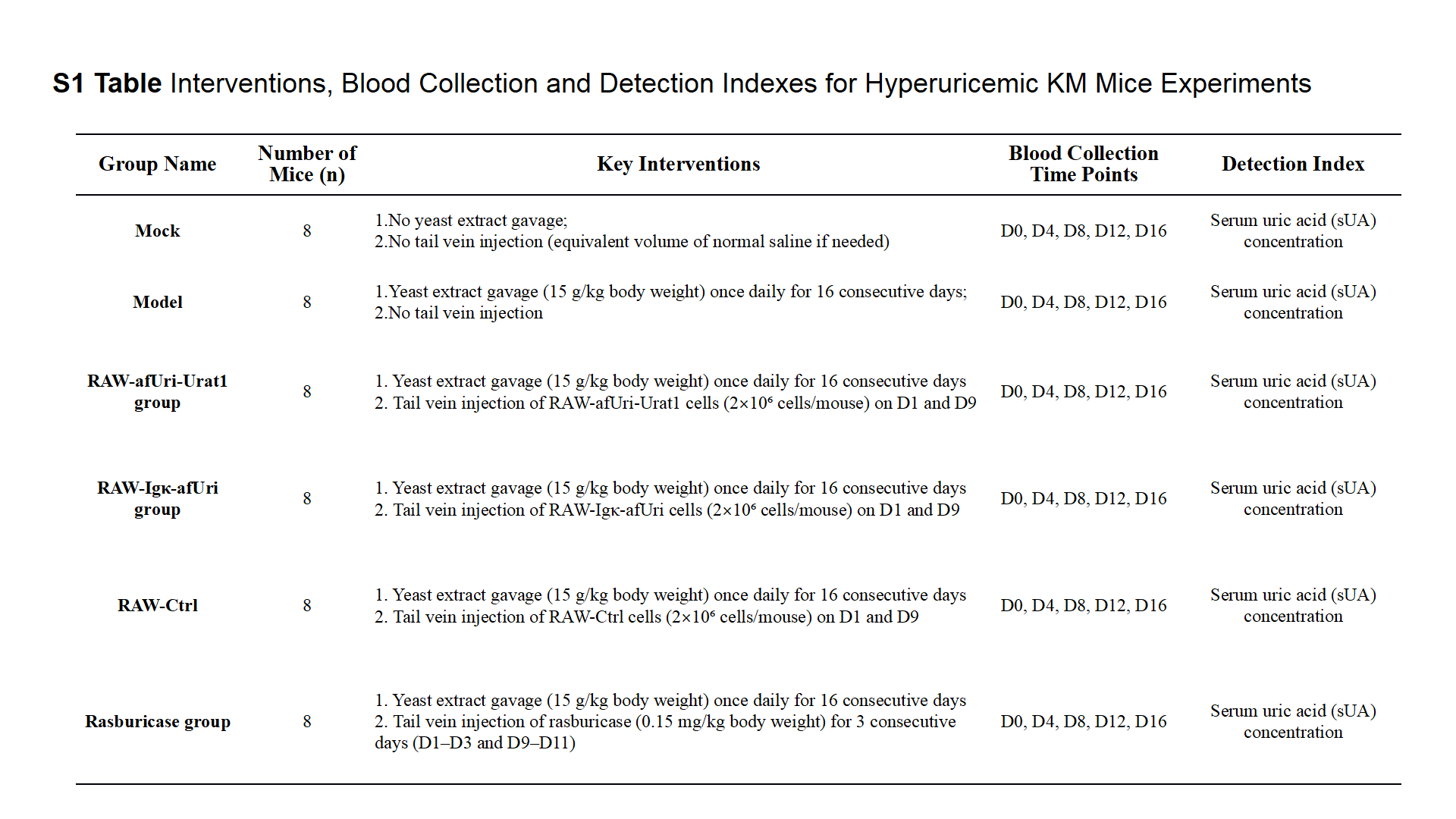

Supplement: S1 Table — (TIF) [file pone.0347534.s006.tif]

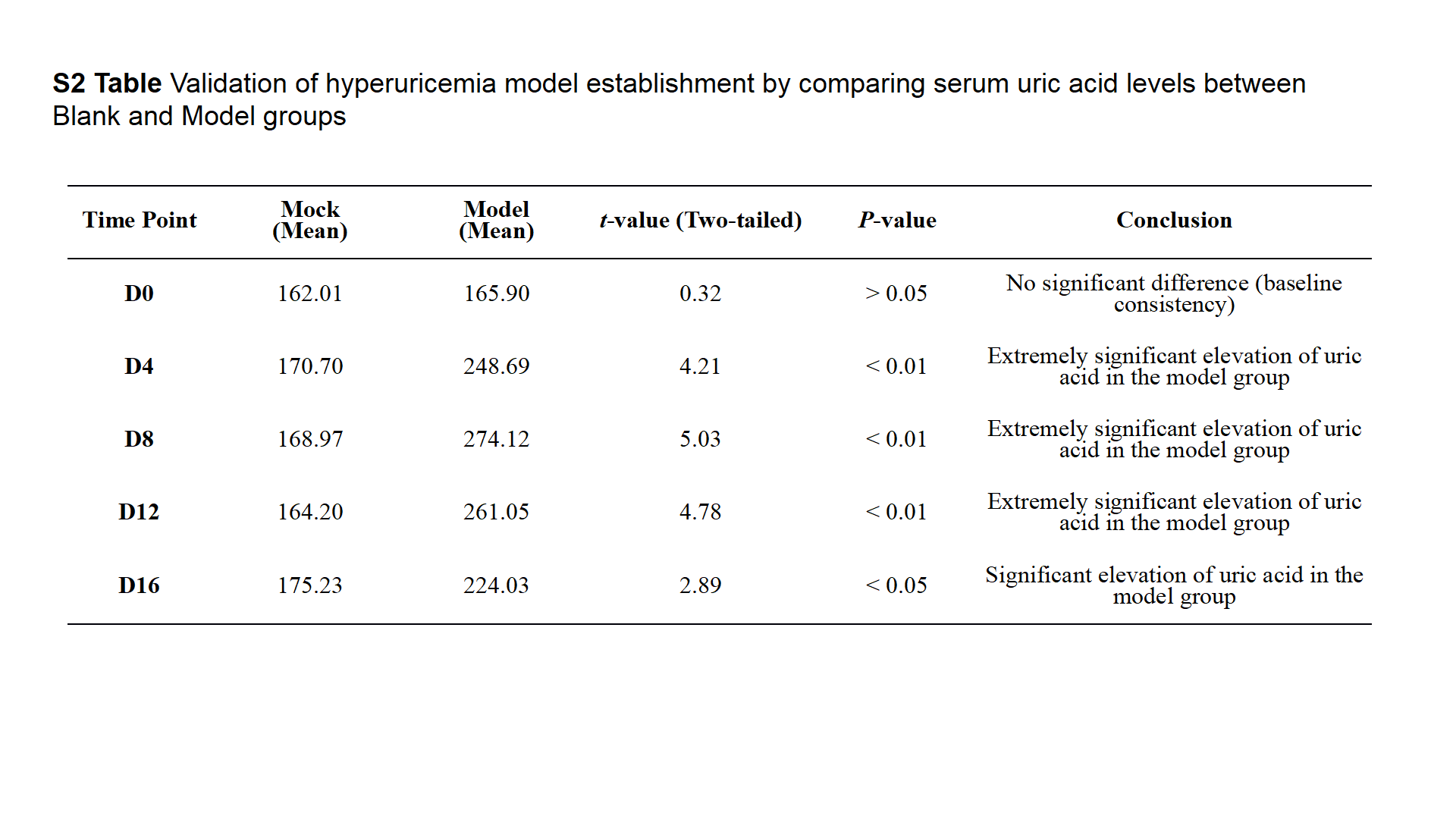

Supplement: S2 Table — (TIF) [file pone.0347534.s007.tif]

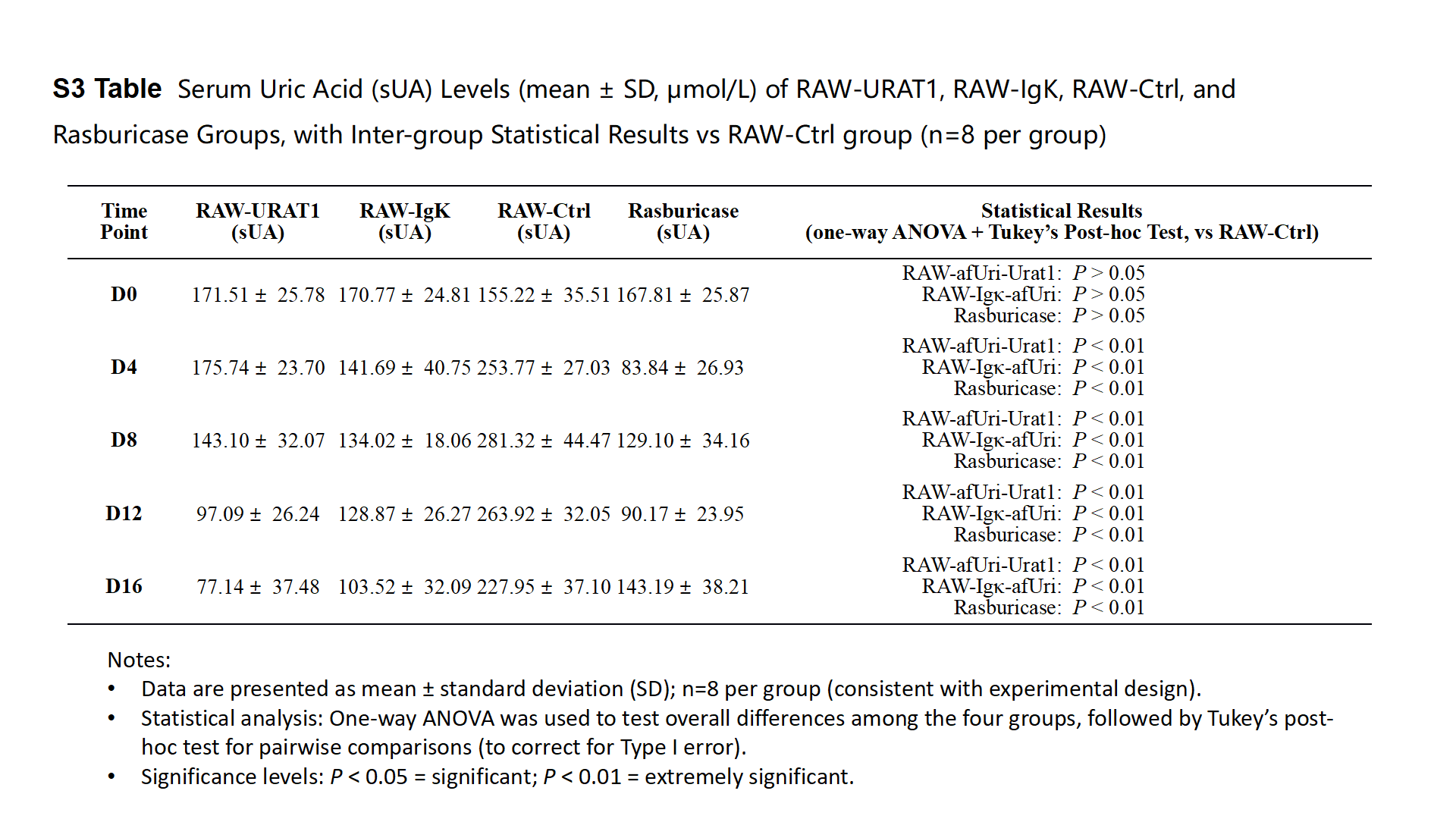

Supplement: S3 Table — (TIF) [file pone.0347534.s008.tif]

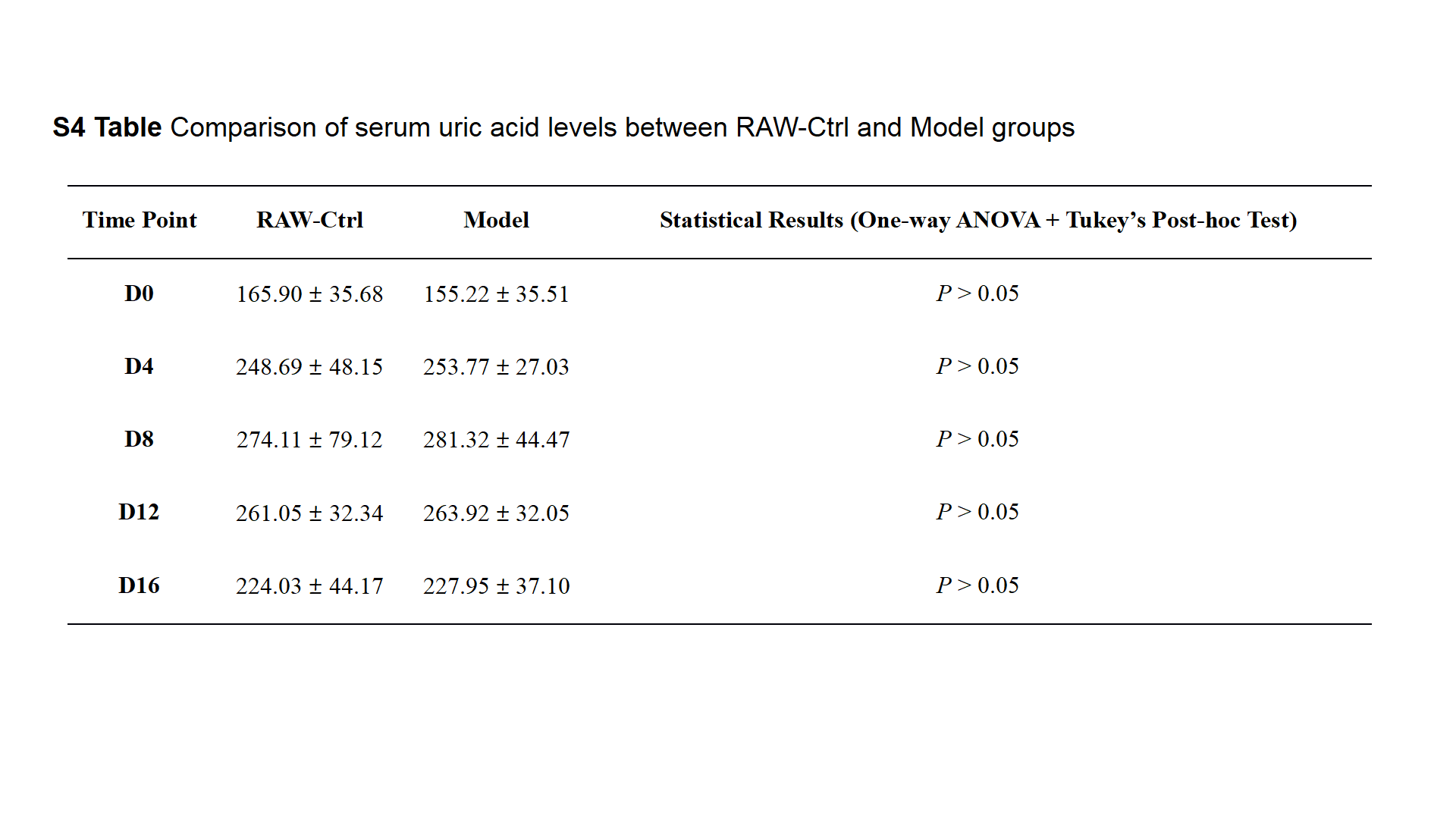

Supplement: S4 Table — (TIF) [file pone.0347534.s009.tif]

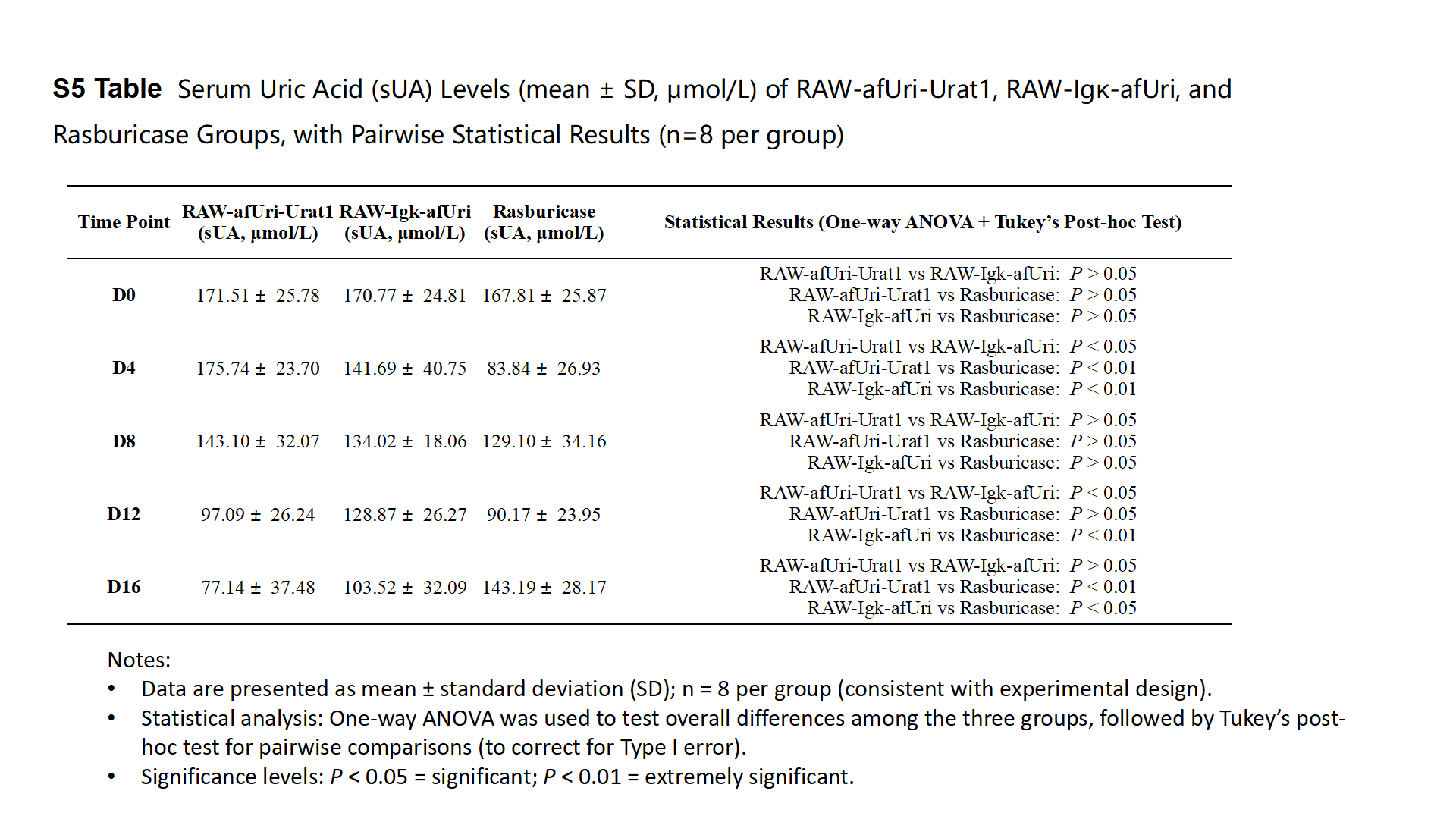

Supplement: S5 Table — (TIF) [file pone.0347534.s010.tif]
