# Supplementary material for: Ectopic expression of Aspergillus flavus uricase and URAT1 in therapeutic cells promotes intracellular degradation of uric acid in hyperuricemic mice
Source: PLoS One. 2026 Apr 20;21(4):e0347534. doi: 10.1371/journal.pone.0347534 (PMC13094949; doi:10.1371/journal.pone.0347534)

WB Actin bands for Fig 2D (background subtracted - 10s exposure)

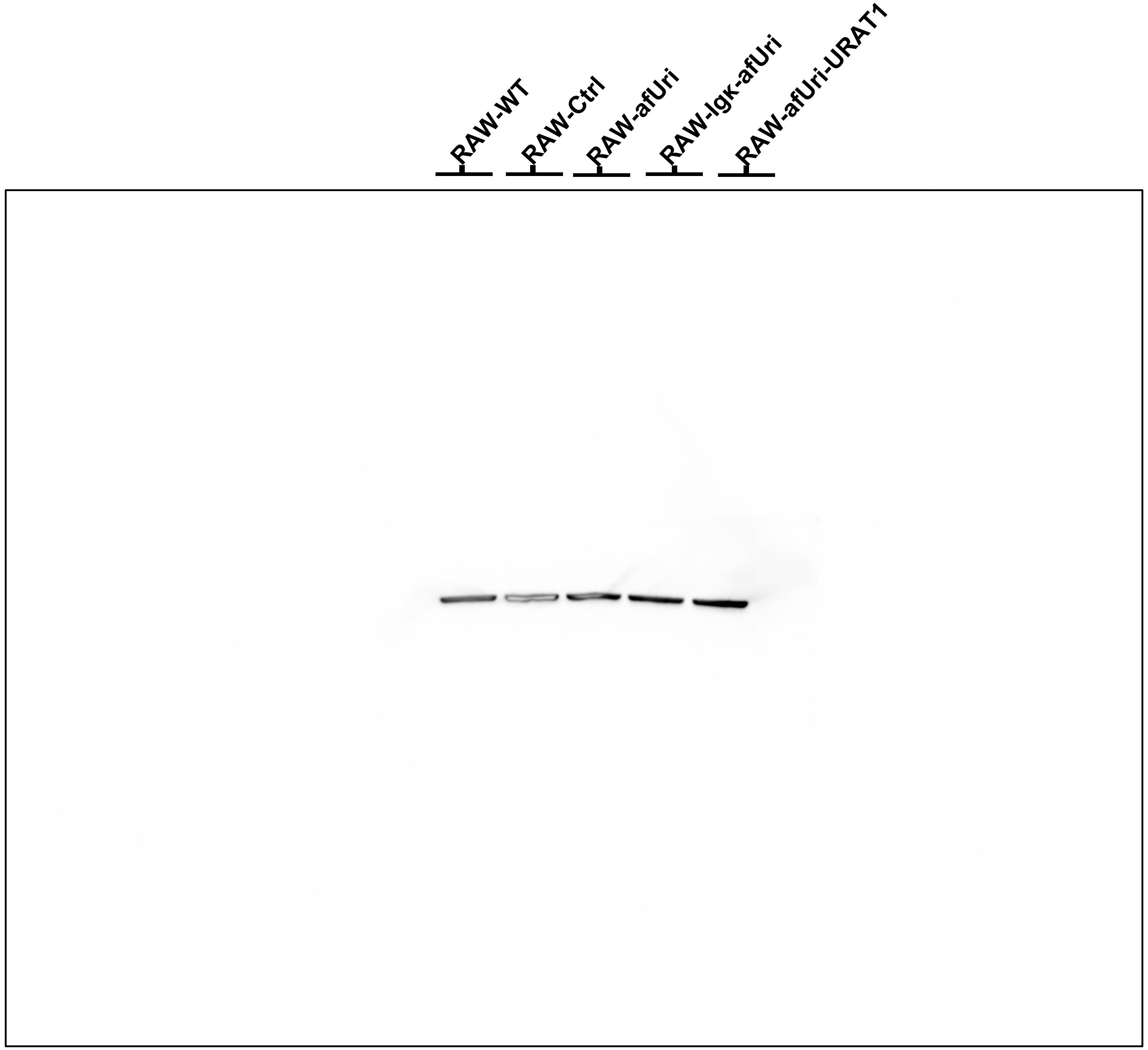

WB Actin bands for Fig 2D (background subtracted - 30s exposure)

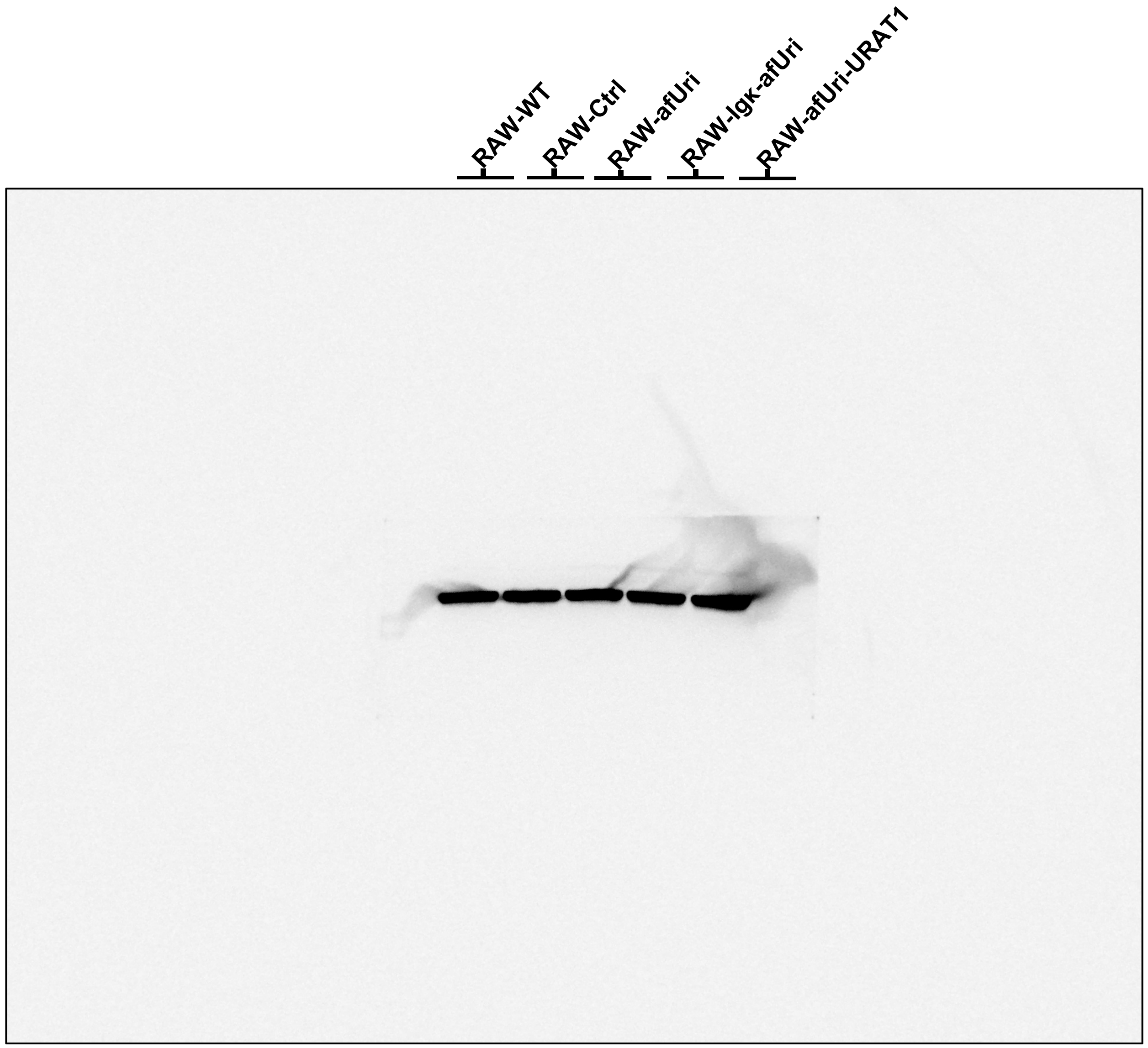

WB Actin bands for Fig 2D (uprocessed - 10s exposure)

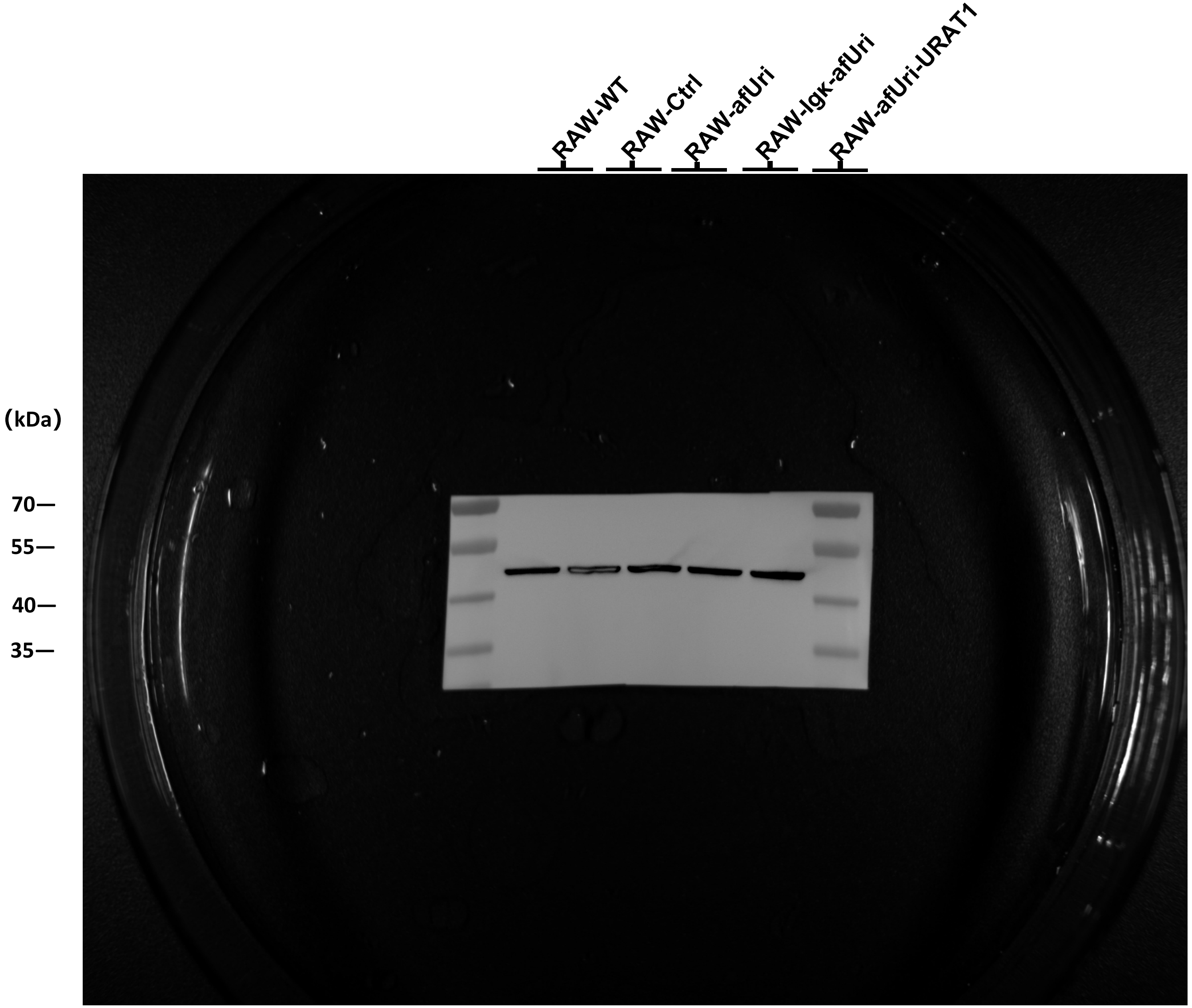

WB Cell FLAG for Fig 2D (background subtracted)

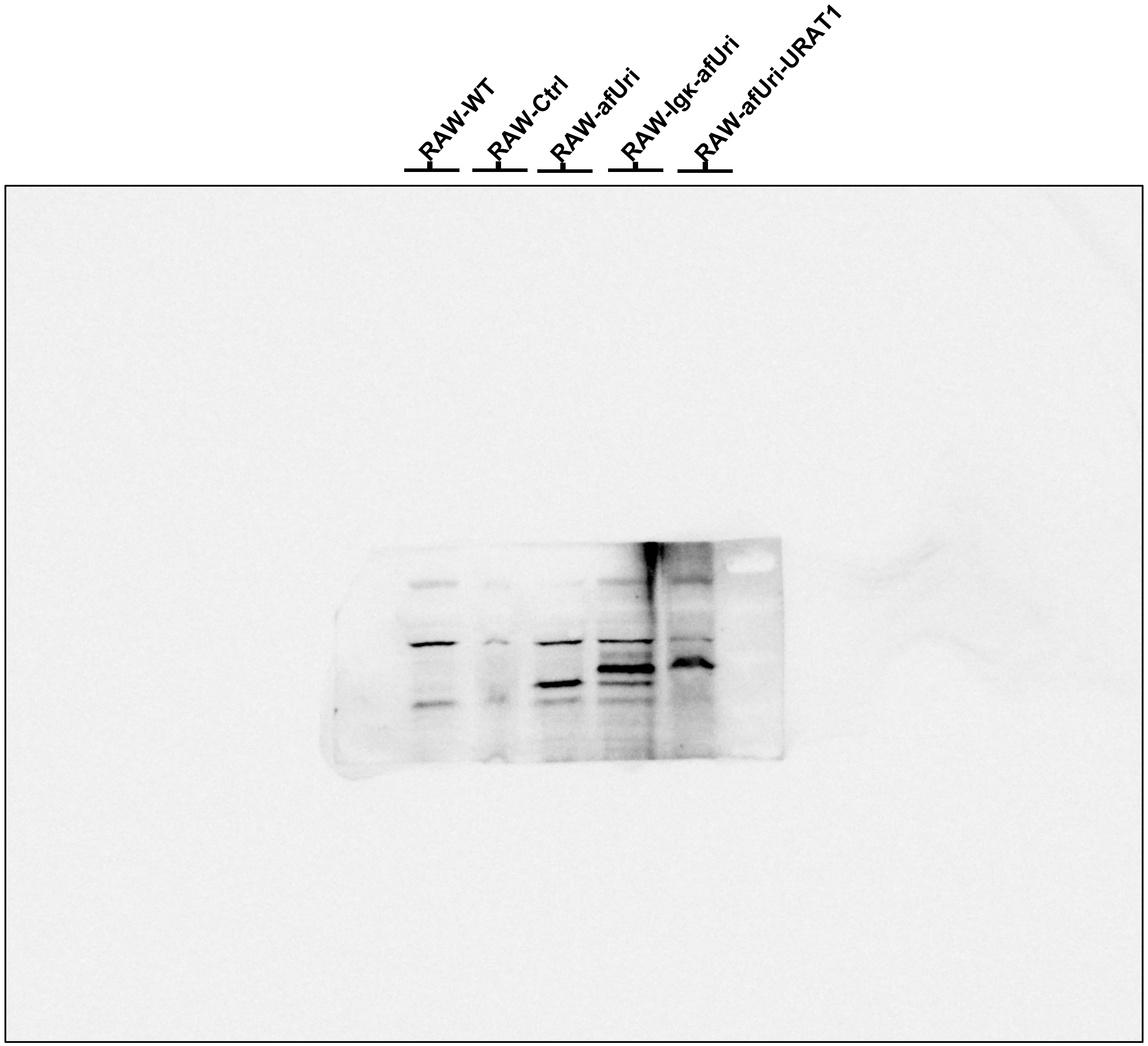

WB Cell FLAG for Fig 2D (unprocessed)

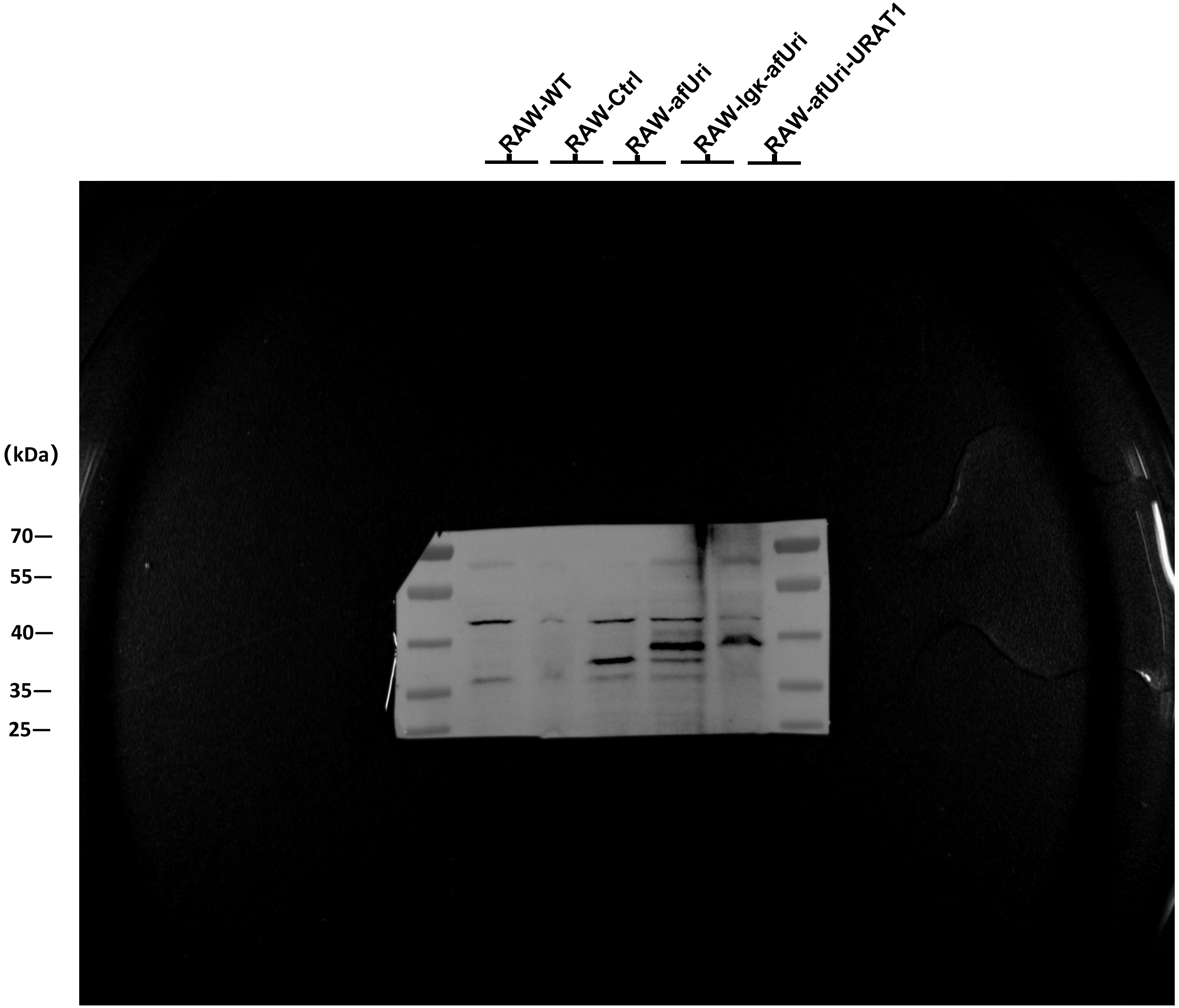

WB Supernatant FLAG for Fig 2E (background subtracted)

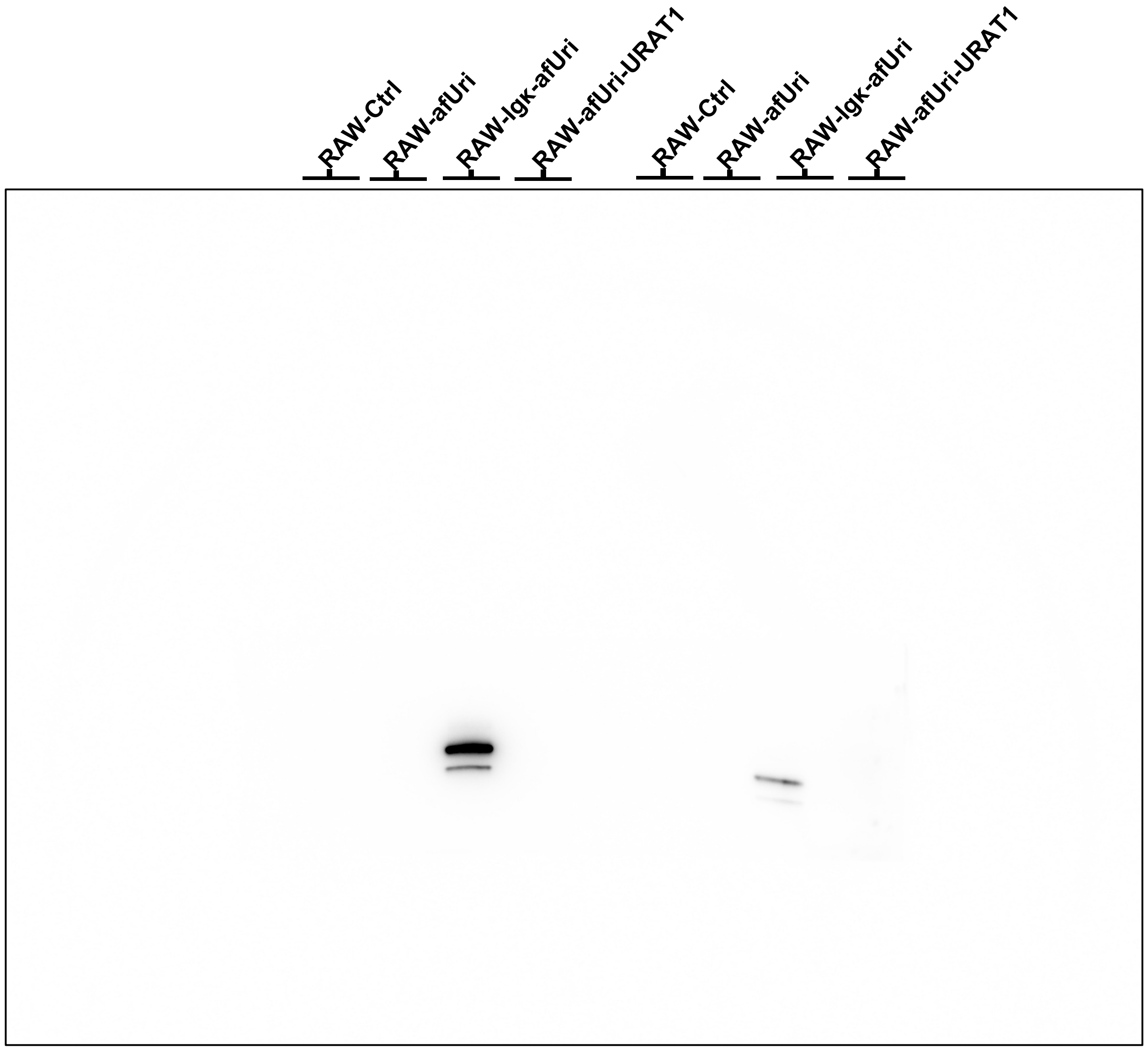

WB Supernatant FLAG for Fig 2E(unprocessed)

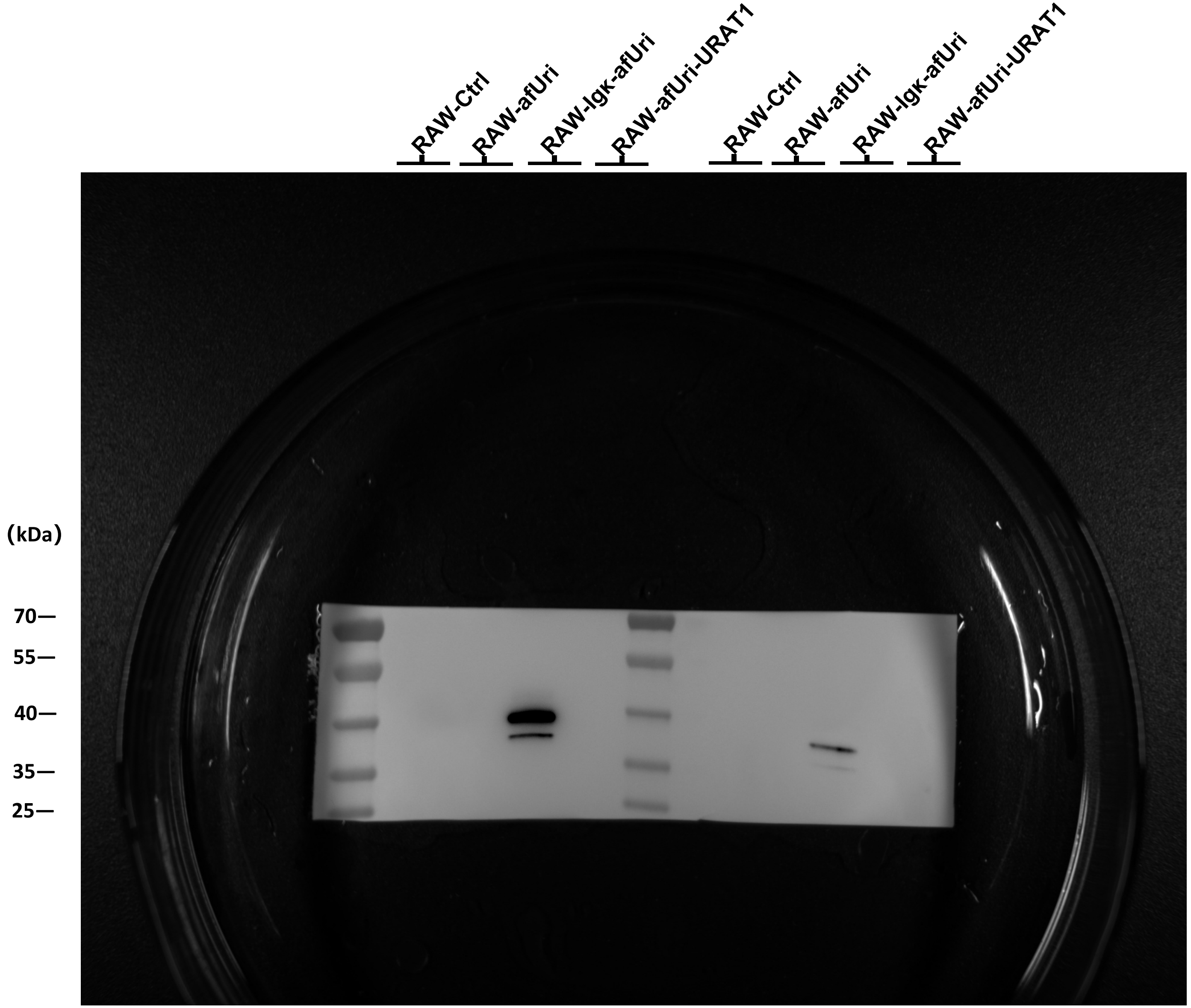

Supplement: S1 File — (PDF) [file pone.0347534.s011.pdf]
