# Supplementary material for: Ectopic expression of Aspergillus flavus uricase and URAT1 in therapeutic cells promotes intracellular degradation of uric acid in hyperuricemic mice
Source: PLoS One. 2026 Apr 20;21(4):e0347534. doi: 10.1371/journal.pone.0347534 (PMC13094949; doi:10.1371/journal.pone.0347534)
Supplement: Attachment — Submitted filename: Response to reviewers.docx [file pone.0347534.s014.docx]

Dear Reviewers,

We greatly appreciate your thorough review and constructive comments, which have significantly helped us improve the quality and rigor of our manuscript. We would like to sincerely note that **the experiments addressing immunogenicity, safety, URAT1 function, and chronic model validation had already been underway and continuously performed before and during the review period**, and we have now fully integrated these data in the revised version.

Below is our point-by-point response to your comments:

**Reply to Reviewer 1:**

### Comment 1: Lack of Direct Immunogenicity Assessment

**The study claims reduced immunogenicity as a key advantage but provides no direct evidence (e.g., measurement of anti-drug antibodies, cytokine levels, or immune cell activation). Relying solely on sustained efficacy as an indirect proxy is insufficient and weakens the translational argument.**

**Response: We sincerely appreciate this critical comment. Given the lack of commercially available standardized ELISA kits for quantitative detection of anti-uricase antibodies, we established two semi-quantitative indirect ELISA assays using rasburicase and secreted *A. flavus* uricase (afUri) as capture antigens, respectively. Although semi-quantitative, these results clearly demonstrate that intracellularly confined uricase in RAW-afUri-URAT1, compared with secreted uricase, did not elicit detectable antibody responses in mice, validating low-immunogenicity advantage. The corresponding results have been added to the Results section as Fig 7.**

### Comment 2: Inadequate Demonstration of URAT1 Functional Activity.

### While URAT1 expression is confirmed, no functional validation (e.g., UA uptake assays, URAT1 inhibition studies, or competitive transport experiments) is provided. Without this, the proposed "transport-degradation" mechanism remains speculative rather than proven.

**Response:** Thank you for this important suggestion. Standard uric acid uptake assays are difficult to perform in our system because **intracellular uricase rapidly degrades transported uric acid**, making direct uptake measurement unreliable. To overcome this limitation, we performed a **URAT1 inhibition assay** using benzbromarone and lesinurad. The results showed that **pharmacological inhibition of URAT1 completely abolished uric acid degradation** by RAW-afUri-URAT1 cells. This functional experiment indicates that **URAT1‑mediated urate uptake is indispensable** for the intracellular degradation mechanism, further validating the function of URAT1 in our engineered system. The data has been added to the Results section as Fig. 4E

### Comment 3: Limited In Vivo Safety and Biodistribution Data.

### The study lacks essential preclinical safety data, including: Biodistribution and persistence of engineered macrophages Potential off-target effects or organ toxicity Inflammatory or immune responses in tissues This omission is significant for a cell-based therapeutic approach moving toward clinical translation.

**Response:**We fully agree with your concern and have supplemented comprehensive safety data in the revision.

**Safety assessment**: We have added **serum biochemistry (liver and renal function)** and **key inflammatory cytokines (IL‑1β, IL‑6, TNF‑α)** detection. All indicators remained within normal physiological ranges, and acute hyperuricemia‑related inflammation was significantly alleviated, confirming good biosafety (S4 Fig, S5 Fig).

**Biodistribution**: The distribution of RAW264.7 cells in the body has been studied by other groups. Sapach *et al*. (2022, DOI: 10.1021/acsami.2c12004) have demonstrated that intravenously administered RAW264.7 cells were mainly accumulated in the liver under physiological conditions. We cited this paper and commented this issue in the Discussion section.

### Comment 4: Use of a Suboptimal Animal Model for Long-Term Evaluation

**Response: We greatly appreciate your professional comment. We also performed therapeutic experiments based on a liver uricase conditional knockout mice established by Pang *et al.* (2024, DOI: 10.1016/j.bbadis.2023.167009.). Unlike mice in gavage model, uricase liver-conditional knockout mice were unable to compensatory upregulate uricase expression as the gene has been knocked out. The data was not included in the initial manuscript because of not enough scale for animal experiments. During the under-review and revision periods, we have completed relative experiments. Our data showed that the urate‑lowering efficacy by RAW‑afUri‑URAT1 sustained up to 28 days, suggesting good durability. We objectively note that 28 days still represents a limited observation period for lifelong clinical translation, and longer‑term evaluation will be included in our future studies. The data is presented as Fig. 6.**

**Reply to Reviewer 2:**

We thank you for your kind and encouraging comments. We appreciate your recognition of the technical soundness, experimental rigor, and proper data presentation of our study. We will further refine the manuscript to align with the journal’s standards.
